# Three-Dimensional Terahertz Coded-Aperture Imaging Based on Matched Filtering and Convolutional Neural Network

**DOI:** 10.3390/s18051342

**Published:** 2018-04-26

**Authors:** Shuo Chen, Chenggao Luo, Hongqiang Wang, Bin Deng, Yongqiang Cheng, Zhaowen Zhuang

**Affiliations:** School of Electronic Science, National University of Defense Technology, Changsha 410073, China; chenshuo13@nudt.edu.cn (S.C.); dengbin@nudt.edu.cn (B.D.); yqcheng@nudt.edu.cn (Y.C.); zwzhuang@nudt.edu.cn (Z.Z.)

**Keywords:** coded-aperture imaging, terahertz, three-dimensional (3D), matched filtering (MF), convolutional neural network (CNN)

## Abstract

As a promising radar imaging technique, terahertz coded-aperture imaging (TCAI) can achieve high-resolution, forward-looking, and staring imaging by producing spatiotemporal independent signals with coded apertures. However, there are still two problems in three-dimensional (3D) TCAI. Firstly, the large-scale reference-signal matrix based on meshing the 3D imaging area creates a heavy computational burden, thus leading to unsatisfactory efficiency. Secondly, it is difficult to resolve the target under low signal-to-noise ratio (SNR). In this paper, we propose a 3D imaging method based on matched filtering (MF) and convolutional neural network (CNN), which can reduce the computational burden and achieve high-resolution imaging for low SNR targets. In terms of the frequency-hopping (FH) signal, the original echo is processed with MF. By extracting the processed echo in different spike pulses separately, targets in different imaging planes are reconstructed simultaneously to decompose the global computational complexity, and then are synthesized together to reconstruct the 3D target. Based on the conventional TCAI model, we deduce and build a new TCAI model based on MF. Furthermore, the convolutional neural network (CNN) is designed to teach the MF-TCAI how to reconstruct the low SNR target better. The experimental results demonstrate that the MF-TCAI achieves impressive performance on imaging ability and efficiency under low SNR. Moreover, the MF-TCAI has learned to better resolve the low-SNR 3D target with the help of CNN. In summary, the proposed 3D TCAI can achieve: (1) low-SNR high-resolution imaging by using MF; (2) efficient 3D imaging by downsizing the large-scale reference-signal matrix; and (3) intelligent imaging with CNN. Therefore, the TCAI based on MF and CNN has great potential in applications such as security screening, nondestructive detection, medical diagnosis, etc.

## 1. Introduction

Recently, electromagnetic (EM) imaging has been widely studied, and many new cutting-edge imaging methods have been proposed, one of which is terahertz coded-aperture imaging (TCAI) [1,2,3]. Different from synthetic aperture radar, TCAI can overcome the difficulties of forward-looking and staring imaging with high resolution. This imaging technology can be seen as an application example of computational imaging (CI) [4,5]. Modern CI schemes provide the mathematical foundation for TCAI that can take advantage of arbitrary measurement modalities, which can be achieved by modulating the signal phase or amplitude randomly with the coded apertures. Based on the basic principles of optical coded-aperture imaging [6,7] and radar coincidence imaging (RCI) [8,9], TCAI adopts an electrical dynamic aperture to either measure or produce spatiotemporal independent signals in the imaging area and then resolve the target scatters by using the pseudo-randomness of the signals. Moreover, terahertz waves (0.1–10 THz) hold many advantages such as stronger penetration capability over light and higher resolution over microwave, allowing visualization of hidden objects at the millimeter level [10,11,12].

Fortunately, metasurfaces have shown great promise in flexible manipulation on terahertz and millimeter waves, which have been applied to areas of high-resolution computational imaging [13,14] and some scanning devices [15,16]. The Harvard Robotics Laboratory (HRL) participated in the advanced scanning technology for imaging radars (ASTIR) project [17] and developed a high-resolution, low-power coded aperture subreflector array (CASA) that can potentially see weapons or explosives concealed on a person at tactically safe distances [18]. Therefore, metasurfaces have the potential for successful integetration in the design of coded apertures for the TCAI system.

However, there are still two problems for 3D TCAI. Due to the large amount of meshed gird cells [3,19], the large-scale reference-signal matrix creates high computational complexity. The other problem is a lack of ability to reconstruct the low-SNR target. Under excessively low SNR, the useful signal is drowned in the noise, and thus leads to the mismatch between the back signal and reference-signal matrix.

In this paper, we propose a new TCAI method based on matched filtering (MF) [20,21] and convolutional neural network (CNN) [22,23,24]. By MF operation on the back signal, the signal-to-noise ratio (SNR) is improved and the spike pulses corresponding to different imaging planes are divided and extracted. Then, the three-dimensional (3D) target reconstruction is decomposed into the combination of two-dimensional (2D) imaging with high SNR. As for CNN, the back signals under different SNRs are generated for training while multiple convolutional layers are designed for learning. The trained CNN, beyond a noise filter, can better teach the MF-TCAI to resolve the low SNR target.

This paper is organized as follows. In Section 2, the basic imaging principle and model of the TCAI is introduced. Based on MF, we describe the creative model and principle of MF-TCAI, the imaging procedure of which is presented in Algorithm 1. In Section 3, CNN is designed for further imaging on the basis of MF-TCAI. Then, we describe the designed CNN architecture, preparation of training and validation sets, followed by the detailed training technique. In Section 4, numerical experiments are conducted to demonstrate the imaging ability of the CNN based MF-TCAI for low SNR 3D targets. Finally, we summarize the main advantages of our architecture and conclude with future directions in Section 5.
**Algorithm 1.** Imaging procedure of matched filtering (MF)-terahertz coded-aperture imaging (TCAI) (MF-TCAI)**Input**The original back-signal vector Sr.**Step 1**Obtain the range-profile vector $Sr_{h}$ by MF process with Sr.**Step 2**Extract $Sr_{h}^{1}$, $Sr_{h}^{2}$, $Sr_{h}^{3}$, and $Sr_{h}^{4}$ of different imaging planes from $Sr_{h}$, and index the related rows, respectively.**Step 3**Construct the MF reference-signal matrices $S_{h}^{1}$, $S_{h}^{2}$, $S_{h}^{3}$, and $S_{h}^{4}$ corresponding to $Sr_{h}^{1}$, $Sr_{h}^{2}$, $Sr_{h}^{3}$, and $Sr_{h}^{4}$, respectively. The constructing operation is shown as:(1)Build the time-domain reference-signal matrices $S_{1}$, $S_{2}$, $S_{3}$, and $S_{4}$ of different imaging planes according to Equations (4) and (5).(2)Obtain the original MF reference-signal matrices $S_{h}^{o1}$, $S_{h}^{o2}$, $S_{h}^{o3}$, and $S_{h}^{o4}$ by MF process with $S_{1}$, $S_{2}$, $S_{3}$, and $S_{4}$, respectively, according to Equation (11).(3)Extract the required $S_{h}^{1}$, $S_{h}^{2}$, $S_{h}^{3}$, and $S_{h}^{4}$ from $S_{h}^{o1}$, $S_{h}^{o2}$, $S_{h}^{o3}$, and $S_{h}^{o4}$ , respectively, referring to the indexed row numbers in Step 2.**Step 4**Reconstruct ${\overset{⌢}{\beta }}_{1}$, ${\overset{⌢}{\beta }}_{2}$, ${\overset{⌢}{\beta }}_{3}$, and ${\overset{⌢}{\beta }}_{4}$ of different imaging planes according to Equation (12).**Output**Return three-dimensional (3D) imaging result $\overset{⌢}{\beta }$ in combination of ${\overset{⌢}{\beta }}_{1}$, ${\overset{⌢}{\beta }}_{2}$, ${\overset{⌢}{\beta }}_{3}$, and ${\overset{⌢}{\beta }}_{4}$.


## 2. Principle and Model

### 2.1. Conventional TCAI

**Figure 1:**
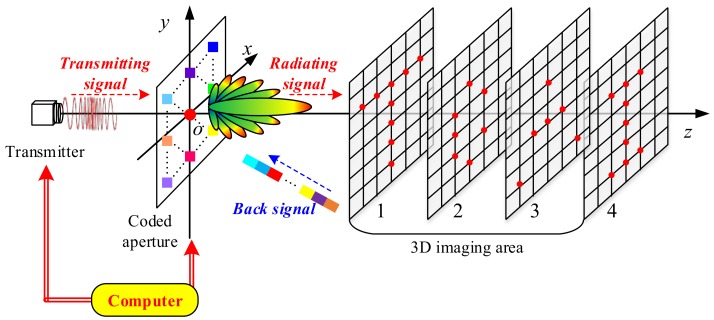
Schematic diagram of 3D TCAI.

As shown in the schematic diagram in Figure 1, the 3D TCAI is mainly composed of a computer, a transmitter, a coded aperture, and the 3D imaging area. The transmitter sends signals in the Terahertz band. The coded aperture, controlled by the computer, can modulate the amplitude or phase of the transmitting signals. On the coded aperture, the colorful squares show the transmitting array elements while the central red point describes the single detector. Different colors on the transmitting array denote different amplitude or phase modulation on the transmitting signal. The 3D imaging area is first subdivided into several imaging planes in different ranges, and then further divided into tiny grid cells, while scatters are assumed to be at the center of the grid cells.

The imaging procedure includes two steps, namely, the transmitting and receiving processes, which are marked as red and blue dashed lines in Figure 1. In the transmitting process, the coded aperture modulates the transmitting signal in a random style, which guarantees successful imaging under TCAI architecture. After being reflected from the 3D imaging area, the signal comes into the receiving process. Eventually, the single detector on the coded aperture receives the back signals and transfers them to the computer for signal processing. Additionally, the computer can control the transmitter and the coded aperture simultaneously. Next, according to Figure 1, we deduce the conventional TCAI imaging model in detail.

#### 2.1.1. The Transmitting Process

Firstly, the transceiver transmits a terahertz frequency-hopping (FH) signal, which is shown as:

(1) $$St(t)=\sum_{m=1}^{M}\mathrm{rect}\left(\frac{t-(m-1)T_{p}}{T_{p}}\right)\cdot A\cdot \exp\left[j2\pi \left(f_{c}+f_{m}\right)t\right]$$

where St(t) is the transmitting FH signal at time t, M, $T_{p}$, and $f_{m}$ are the number, width, and hopping frequency of the sub-pulse, respectively. A is the amplitude, $f_{c}$ is the center frequency, and rect(·) is the rectangular window function.

For convenience of clarity, we assume the FH signal arriving at the coded aperture is a plane wave. The time delay terms for each transmitting element of the coded aperture are the same, and they can be set as zero. As the coded aperture contains *I* transmitting elements, the radiating signal through the coded aperture can be expressed as:

(2) $$S_{c}(t)=\sum_{i=1}^{I}\sum_{m=1}^{M}\mathrm{rect}\left(\frac{t-(m-1)T_{p}}{T_{p}}\right)\cdot A_{t,i}\cdot \exp\left[j2\pi \left(f_{c}+f_{m}\right)t\right]\cdot \exp\left(j\cdot \varphi_{t,i}\right)$$

where $A_{t,i}$ and $\varphi_{t,i}$ are the random modulation terms of amplitude and phase for the *i*-th transmitting element at time t, respectively.

Then, the radiating signal illustrates the 3D imaging area, which contains K grid cells. For high-resolution imaging, the radiation field on the 3D imaging area is spatiotemporally independent.

#### 2.1.2. The Receiving Process

Reflected by the 3D target, the back signal arriving at the receiving element of the coded aperture is denoted as:

(3) $$Sr(t)=\sum_{k=1}^{K}\sum_{i=1}^{I}\sum_{m=1}^{M}\mathrm{rect}\left(\frac{t-(m-1)T_{p}-t_{i,k}}{T_{p}}\right)\cdot A_{t,i}\cdot \beta_{k}\cdot \exp\left[j2\pi \left(f_{c}+f_{m}\right)\left(t-t_{i,k}\right)\right]\cdot \exp\left(j\cdot \varphi_{t,i}\right)$$

where $\beta_{k}$ is the scattering coefficient corresponding to the *k*-th grid-cell, $t_{i,k}$ is the total time delay passing though the *i*-th transmitting element, the *k*-th grid cell, and the receiving element of the coded aperture.

Based on the time discretion of Equation (3), the conventional mathematical model of TCAI can be formulated as:

(4) $$\begin{array}{l} \mathrm{Sr}=S\cdot \beta \\ \left[\begin{array}{c} Sr\left(t_{1}\right)\\ Sr\left(t_{2}\right)\\ \cdots \\ Sr\left(t_{N}\right) \end{array}\right]=\left[\begin{array}{cccc} S\left(t_{1},1\right) & S\left(t_{1},2\right) & \cdots & S\left(t_{1},K\right)\\ S\left(t_{2},1\right) & S\left(t_{2},2\right) & \cdots & S\left(t_{2},K\right)\\ \cdots & \cdots & \cdots & \cdots \\ S\left(t_{N},1\right) & S\left(t_{N},2\right) & \cdots & S\left(t_{N},K\right) \end{array}\right]\cdot \left[\begin{array}{c} \beta_{1}\\ \beta_{2}\\ \cdots \\ \beta_{K} \end{array}\right] \end{array}$$

where $\mathrm{Sr}=\left(Sr\left(t_{n}\right)\right),n=1,\cdots N$, $S=\left(S\left(t_{n},k\right)\right),k=1,\cdots K,n=1,\cdots ,N$ and $\beta =\left(\beta_{k}\right),k=1,\cdots K$ are the back-signal vector, reference-signal matrix, and scattering-coefficient vector, respectively. *N* and *K* are the numbers of sampling time and grid cells, respectively. The array element of **S** is

(5) $$S\left(t_{n},k\right)=\sum_{i=1}^{I}\sum_{m=1}^{M}\mathrm{rect}\left(\frac{t-(m-1)T_{p}-t_{i,k}}{T_{p}}\right)\cdot A_{t,i}\cdot \exp\left[j2\pi \left(f_{c}+f_{m}\right)\left(t-t_{i,k}\right)\right]\cdot \exp\left(j\cdot \varphi_{t,i}\right)$$

Using the knowledge of solving linear equations, Equation (4) is difficult to solve when the receiving signal vector **Sr** mismatches the reference-signal matrix **S**. Unfortunately, the reference-signal matrix is deduced from Equation (5) under ideal conditions while the real back signal is received with low SNR.

### 2.2. MF-Based TCAI

To solve the low SNR problem for TCAI, we deduce a new imaging model based on pulse compression by MF. The detailed imaging procedures and model are presented below.

#### 2.2.1. MF Process with the Back-Signal Vector

For the MF process, we define a local-oscillator signal, which is written as:

(6) $$S_{local}(t)=\sum_{m=1}^{M}\mathrm{rect}\left(\frac{t-(m-1)T_{p}}{T_{p}}\right)\cdot \exp\left[j2\pi f_{c}t\right]$$

By mixing the back signal and the local-oscillator signal, the baseband back signal is defined as:

(7) $$\begin{array}{ll} Sr_{base}(t) & =Sr(t)\cdot S_{local}^{*}(t)\\ & =\sum_{k=1}^{K}\sum_{i=1}^{I}\sum_{m=1}^{M}\begin{array}{l} \mathrm{rect}\left(\frac{t-(m-1)T_{p}-t_{i,k}}{T_{p}}\right)\cdot A_{t,i}\cdot \beta_{k}\\ \cdot \exp\left\{j2\pi \left[f_{m}\left(t-t_{i,k}\right)-f_{c}t_{i,k}\right]\right\}\cdot \exp\left(j\cdot \varphi_{t,i}\right) \end{array} \end{array}$$

According to Equation (1), the baseband transmitting signal is formulated as:

(8) $$St_{base}(t)=\sum_{m=1}^{M}\mathrm{rect}\left(\frac{t-(m-1)T_{p}}{T_{p}}\right)\cdot \exp\left(j2\pi f_{m}t\right)$$

To obtain the matched filter, it is required to inverse the time of $St_{base}(t)$ and take its complex conjugate. In this way, the matched filter h(t) is deduced as:

(9) $$h(t)=\sum_{m=1}^{M}\mathrm{rect}\left(\frac{t+(m-1)T_{p}}{T_{p}}\right)\cdot \exp\left(j2\pi f_{m}t\right)$$

Then, the baseband back signal $Sr_{base}(t)$ convolves with the matched filter h(t). The detailed operation is deduced below:

(10) $$\begin{array}{ll} Sr_{h}(t) & =Sr_{base}⊗h(t)\\ & =\int_{-\infty }^{\infty }\sum_{k=1}^{K}\sum_{i=1}^{I}\sum_{m=1}^{M}\begin{array}{l} \mathrm{rect}\left(\frac{u-(m-1)T_{p}-t_{i,k}}{T_{p}}\right)\cdot A_{i}\cdot \beta_{k}\cdot \exp\left\{j2\pi \left[f_{m}\left(u-t_{i,k}\right)-f_{c}t_{i,k}\right]\right\}\cdot \exp\left(j\cdot \varphi_{t,i}\right)\\ \cdot \sum_{l=1}^{M}\mathrm{rect}(\frac{t-u+(l-1)T_{p})}{T_{p}})\cdot \exp[j2\pi f_{l}(t-u)]du \end{array}\\ & =\sum_{k=1}^{K}\sum_{i=1}^{I}\sum_{m=1}^{M}\sum_{l=1}^{M}\begin{array}{l} A_{i}\cdot \beta_{k}\cdot \exp[j2\pi t_{t,k}[f_{m}+f_{c}]]\cdot \exp(j2\pi f_{l}t)\cdot \exp(j\cdot \varphi_{t,i})\\ \cdot \int_{-\infty }^{\infty }\mathrm{rect}(\frac{u-(m-1)T_{p}-t_{i,k}}{T_{p}})\cdot \mathrm{rect}\frac{t-u+(l-1)T_{p}}{T_{p}}\exp[j2\pi u(f_{m}-f_{l})]du \end{array}\\ & =\sum_{k=1}^{K}\sum_{i=1}^{I}\sum_{m=1}^{M}\sum_{l=1}^{M}A_{i}\cdot \beta_{k}\cdot \exp\{j\varphi \}\cdot (T_{p}-|\tau |)\cdot \mathrm{rect}(\frac{\tau }{2T_{p}})\cdot \sin c[\pi v(f_{m}-f_{l})] \end{array}$$

where $\varphi =2\pi t_{i,k}\left(f_{m}+f_{c}\right)+2\pi f_{l}t+\varphi_{t,i}$ and $\tau =t-\left(m-1\right)T_{p}+\left(l-1\right)T_{p}-t_{ik}$. In observation of Equation (10), the MF output signal relies mainly on the functions of rect(·) and sinc(·). $\sin c\left(v\right)=\frac{\sin\left(\pi v\right)}{\pi v}$ is a pulse function.

#### 2.2.2. Extraction of the Range-Profile Vector

Herein, we define the range-profile vector as $Sr_{h}$, which is described in Equation (10). Because of the function property of rect(·) and sinc(·), Equation (10) presents spike pulses at m=l and $t=t_{ik}$, where $t_{i,k}=r_{i,k}/c$, and $r_{i,k}$ is the distance delay corresponding to $t_{i,k}$. Therefore, the scattering information within the same range gathers in the same spike pulse.

The 3D imaging area in Figure 1 has four imaging planes in different ranges. As each imaging plane is in about a one range cell, the range-profile vector $Sr_{h}$ will show four spike pulses. On the one hand, the four spike pulses include all the information of the 3D target. On the other hand, each spike pulse only contains the target information within the same imaging plane. By extracting the corresponding spike pulse of $Sr_{h}$, the targets in different imaging planes can be reconstructed one by one, and then can be synthesized together to obtain the whole 3D target.

**Figure 2:**
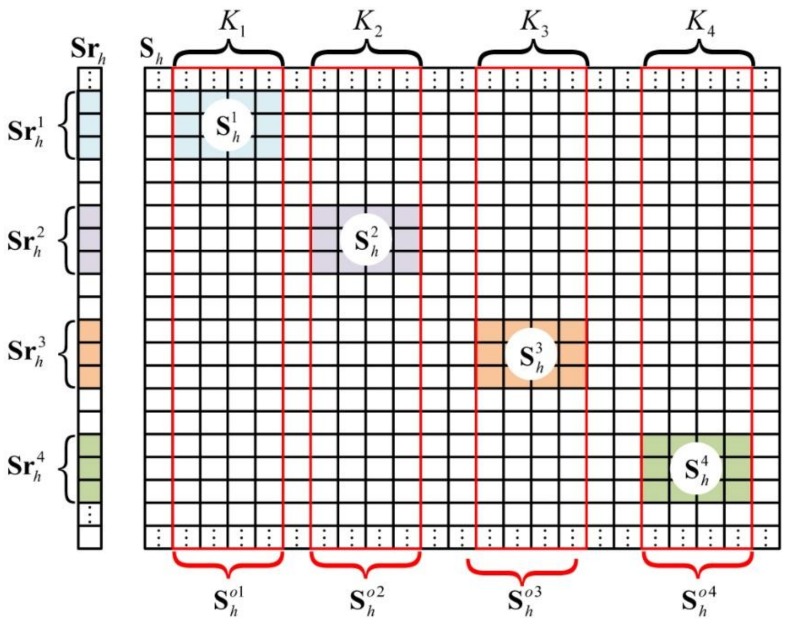
Extraction of the range-profile vector and conformation of the reference-signal matrix.

To construct the new range-profile vectors, different spike pulses in $Sr_{h}$ are extracted separately. For example, the $Sr_{h}$ from Figure 1 can be subdivided into $Sr_{h}^{1}$, $Sr_{h}^{2}$, $Sr_{h}^{3}$, and $Sr_{h}^{4}$, which are shown in Figure 2. Moreover, $r_{1}$, $r_{2}$, $r_{3}$, and $r_{4}$ are indexed as the corresponding row positions of $Sr_{h}^{1}$, $Sr_{h}^{2}$, $Sr_{h}^{3}$, and $Sr_{h}^{4}$, respectively, in $Sr_{h}$.

#### 2.2.3. Construction of the MF Reference-Signal Matrix

As shown in Figure 2, $K_{1}$, $K_{2}$, $K_{3}$, and $K_{4}$ are the numbers of the grid cells in the four imaging planes, respectively. $S_{h}$ is the MF reference-signal matrix related to $Sr_{h}$. $S_{h}^{1}$, $S_{h}^{2}$, $S_{h}^{3}$, and $S_{h}^{4}$ are the MF reference-signal matrices corresponding to $Sr_{h}^{1}$, $Sr_{h}^{2}$, $Sr_{h}^{3}$, and $Sr_{h}^{4}$, respectively. Moreover, $S_{h}^{1}$, $S_{h}^{2}$, $S_{h}^{3}$, and $S_{h}^{4}$ are extracted partly from $S_{h}^{o1}$, $S_{h}^{o2}$, $S_{h}^{o3}$, and $S_{h}^{o4}$, respectively, which will be further introduced. Instead of the whole matrix $S_{h}$, only $S_{h}^{1}$, $S_{h}^{2}$, $S_{h}^{3}$, and $S_{h}^{4}$ need to be constructed.

Firstly, the time-domain reference-signal matrices $S_{1}$, $S_{2}$, $S_{3}$, and $S_{4}$ are deduced from Equations (4) and (5).

Referring to the process of the back signal in Section 2.2.1, the matched filter h(t) convolves each column of $S_{1}$, $S_{2}$, $S_{3}$, and $S_{4}$. For example, S(t,k), the reference signal in the *k*-th column, is processed with

(11) $$S_{h}\left(t,k\right)=S\left(t,k\right)⊗h\left(t\right)$$

Then, we obtain the original MF reference-signal matrices $S_{h}^{o1}$, $S_{h}^{o2}$, $S_{h}^{o3}$, and $S_{h}^{o4}$.

As shown in Figure 2, the row numbers of $S_{h}^{o1}$, $S_{h}^{o2}$, $S_{h}^{o3}$, and $S_{h}^{o4}$ are the same as $Sr_{h}$. As described in Section 2.2.2, $r_{1}$, $r_{2}$, $r_{3}$, and $r_{4}$ are the row-position tags of $Sr_{h}^{1}$, $Sr_{h}^{2}$, $Sr_{h}^{3}$, and $Sr_{h}^{4}$, respectively, in $Sr_{h}$. Then, we can use $r_{1}$, $r_{2}$, $r_{3}$, and $r_{4}$ to extract corresponding rows of $S_{h}^{o1}$, $S_{h}^{o2}$, $S_{h}^{o3}$, and $S_{h}^{o4}$, respectively, and finally get the required reference-signal matrices $S_{h}^{1}$, $S_{h}^{2}$, $S_{h}^{3}$, and $S_{h}^{4}$.

#### 2.2.4. Target Reconstruction Based on MF

For an imaging plane named *x*, for example, the mathematical model based on MF can be deduced as:

(12) $$\begin{array}{l} {\mathrm{Sr}}_{h}^{x}=S_{h}^{x}\cdot \beta_{x}\\ \left[\begin{array}{c} Sr_{h}\left(t_{1}\right)\\ Sr_{h}\left(t_{2}\right)\\ \cdots \\ Sr_{h}\left(t_{N_{x}}\right) \end{array}\right]=\left[\begin{array}{cccc} S_{h}\left(t_{1},1\right) & S_{h}\left(t_{1},2\right) & \cdots & S_{h}\left(t_{1},K_{x}\right)\\ S_{h}\left(t_{2},1\right) & S_{h}\left(t_{2},2\right) & \cdots & S_{h}\left(t_{2},K_{x}\right)\\ \cdots & \cdots & \cdots & \cdots \\ S_{h}(t_{N_{x}},1) & S_{h}\left(t_{N_{x}},2\right) & \cdots & S_{h}\left(t_{N_{x}},K_{x}\right) \end{array}\right]\cdot \left[\begin{array}{c} \beta_{1}\\ \beta_{2}\\ \cdots \\ \beta_{K_{x}} \end{array}\right] \end{array}$$

where $Sr_{h}^{x}=\left(Sr_{h}\left(t_{n_{x}}\right)\right)$, $S_{h}^{x}=\left(S_{h}\left(t_{n_{x}},k_{x}\right)\right)$, and $\beta_{x}=\left(\beta_{k_{x}}\right)$ are the MF range-profile vector, reference-signal matrix, and scattering-coefficient vector, respectively. Besides, $n_{x}=1,\cdots ,N_{x}$, $k_{x}=1,\cdots K_{x}$, $N_{x}$ and $K_{x}$ are the numbers of sampling time and grid cells, respectively.

Based on Equation (12), each imaging plane in Figure 1 can be reconstructed in parallel to decompose the global computational complexity, and they can then be combined to reconstruct the 3D target.

To make the imaging theory clearer, the MF-TCAI procedure is presented in Algorithm 1.

## 3. Improved Imaging by CNN

### 3.1. CNN Architecture Design

**Figure 3:**
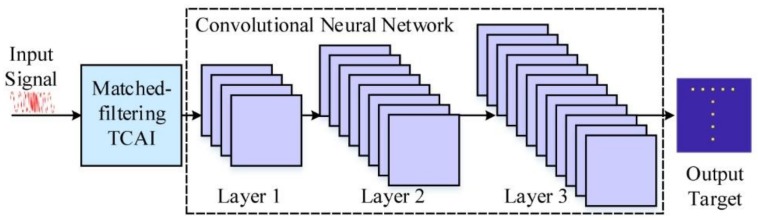
Schematic diagram of MF-TCAI based on convolutional neural network (CNN).

As shown in Figure 3, the improved imaging method includes an MF process and CNN. To reconstruct the primary target, the back signal is fed into the MF-TCAI process, which has been introduced in detail in Section 2.2. The key function of the trained CNN lies in removing the false scattering points and filtering the grain noise of the MF-reconstructed target. Then, for further imaging, the treated target feeds forward into the convolutional neural network (CNN), including but not limited to three convolutional layers, as shown in Figure 3.

**Figure 4:**
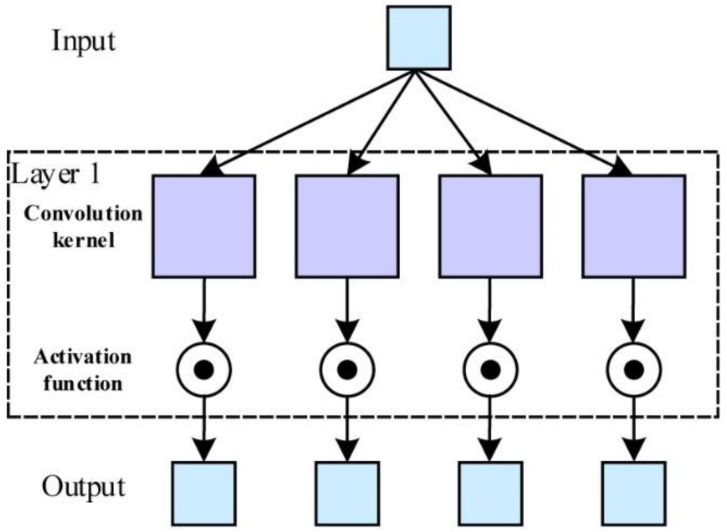
Construction of the non-output layer.

As an example of convolutional layer 1, Figure 4 shows the construction of the non-output layer. As this convolutional layer has one input and four outputs, it needs four convolutional kernels to transform the input image into four feature maps, which can be expressed as:

(13) $$u^{l}=W^{l}x^{l-1}+b^{l}$$

where *l* denotes the *l*-th convolutional layer, $u^{l}$ is the feature map of the layer *l*. $x^{l-1}$ is the output of the layer (*l* − 1), or the input of the layer *l*. $W^{l}$ and $b^{l}$ are the weight coefficient and additive bias of the convolutional kernel, respectively. During the training process, $W^{l}$ and $b^{l}$ become more and more suitable for high-resolution MF-TCAI.

The feature maps in Figure 4, convolved by the kernels, are further processed by the activation function. Our CNN adopts Relu(·) as the activation function, which is shown as:

(14) $$\mathrm{Relu}\left(u\right)=\{\begin{array}{c} u,\;u\geq 0\\ 0,\;u<0 \end{array}$$

where u is the feature-map value.

Different from the non-output layer, the activation function of the output layer is excluded, or it can be interpreted as Relu(u)=u.

### 3.2. CNN Learning Technique

Based on the designed CNN structure, the learning process is described below.

#### 3.2.1. Generation of Training and Validation Sets

Firstly, we set the primary TCAI parameters, such as the transmitting waveform, bandwidth, size and array-element number of the coded aperture, working distance, and the grid-cell generation in the 3D imaging area.

Then, $Nx=Nx_{t}+Nx_{v}$ targets are generated for 3D imaging, where $Nx_{t}$ and $Nx_{v}$ are the target numbers of the training and validation sets, respectively. Generally, Nx and $Nx_{t}:Nx_{v}$ can be set as 50,000–100,000 and 3–4, respectively. During the generation of targets, the number and position of the scattering points in the imaging area are both generated randomly.

Based on the TCAI parameters and Nx targets, Nx echoes are deduced with Gaussian white noises. Then, the $Nx_{t}$ targets and echoes are tagged as the training set, while the other targets and echoes are labeled as validation sets. The training set aims to train the CNN when the validation set is used to test its training effect without participation in the training process.

#### 3.2.2. CNN Parameters Set

The number of convolutional layers is usually set as 3–6. Too many layers will increase the training burden, and easily lead to overfitting, while insufficient layers cannot achieve the learning effect.

The size of the kernel is denoted as Nc×Nc, weight coefficient is $W^{l}$, and additive bias is $b^{l}$. Taking input-size $N_{i}\times N_{i}=30\times 30$ for example, Nc×Nc can be set as 3 × 3, 5 × 5, 7 × 7, etc. Due to the convolutional process, the output-size $N_{o}\times N_{o}$ is reduced to $\left(N_{i}-N_{c}+1\right)\times \left(N_{o}-N_{c}+1\right)$. To keep $N_{o}\times N_{o}$ the same as $N_{i}\times N_{i}$, the input image is expanded to $\left(N_{i}+N_{c}-1\right)\times \left(N_{i}+N_{c}-1\right)$ by adding zeroes around the original image. Moreover, $W^{l}$ and $b^{l}$ are assigned with random numbers from the interval [0,1].

Furthermore, it is required to value the gradient descent coefficient, learning rate, training cycle, and batch size during each training, and so on.

#### 3.2.3. CNN Training Process

In this step, the momentum stochastic gradient descent (SGD) method is used to train $W^{l}$ and $b^{l}$. The training process obeys two learning rules, including the forward and back propagations.

The forward propagation transports the initial reconstruction target from the first to the last convolution layer, and then outputs the final reconstruction result. During the forward propagation, the *l*-th layer outputs

(15) $$x^{l}=f(u^{l})$$

where f(·) is the activation function, which has been introduced in Equation (14). Moreover, **u**^l^ is handled with Equation (13).

The back propagation modifies $W^{l}$ and $b^{l}$ in all layers by using cost function, which is optimized by:

(16) $$E=\frac{1}{2}{\left\|t-y\right\|}_{2}^{2}$$

where E is the cost function, and t and y are the true and output targets, respectively. Then, we can compute the derivatives of E with respect to the network weights $W^{l}$ and $b^{l}$.

Herein, $\delta^{l}=\frac{\partial E}{\partial u^{l}}$ is the sensitivity of the *l*-th layer. As a non-output layer, the sensitivity can be deduced as:

(17) $$\delta^{l}={\left(W^{l+1}\right)}^{T}\delta^{l+1}\circ f^{\prime }(u^{l})$$

where “∘” denotes element-wise multiplication.

Given the index of the output layer as *L*, the sensitivity of which reads

(18) $$\delta^{L}=f^{\prime }(u^{L})\circ (y-t)$$

It is easy to deduce that $\frac{\partial u^{l}}{\partial b^{l}}=1$ from Equation (13), so the bias derivative for layer *l* can be written as:

(19) $$\frac{\partial E}{\partial b^{l}}=\frac{\partial E}{\partial u^{l}}\frac{\partial u^{l}}{\partial b^{l}}=\frac{\partial E}{\partial u^{l}}=\delta^{l}$$

Similarly, from Equation (13), the weight derivative for layer *l* is deduced as:

(20) $$\frac{\partial E}{\partial W^{l}}=x^{l-1}{\left(\delta^{l}\right)}^{T}$$

According to Equations (16)–(20), the cost function error can be back propagated to all layers for updating the weights and biases, which are described as:

(21) $$W_{\mathrm{new}}^{l}=W^{l}-\eta_{1}\cdot \frac{\partial E}{\partial W^{l}}-\eta_{2}\cdot W^{l}$$

(22) $$b_{\mathrm{new}}^{l}=b^{l}-\eta_{1}\cdot \frac{\partial E}{\partial b^{l}}-\eta_{2}\cdot b^{l}$$

where $W_{\mathrm{new}}^{l}$ and $b_{\mathrm{new}}^{l}$ are the updated weight and bias, respectively. $\eta_{1}$ and $\eta_{2}$ are the gradient decent coefficient and learning rate, respectively.

Finally, we can obtain the high-resolution image from the output of the trained CNN. The CNN-learning based MF-TCAI is presented in Algorithm 2.
**Algorithm 2.** Procedure of CNN-learning based MF-TCAI**Input**Initial 3D reconstruction $\overset{⌢}{\beta }$ from MF-TCAI.**Step 1**Generate training and validation sets.**Step 2**Set CNN parameters: the number of convolutional layers *L*, the size of the kernel Nc×Nc, weight coefficient $W^{l}$, additive bias is $b^{l}$, gradient descent coefficient $\eta_{1}$, learning rate $\eta_{2}$, training cycle, and batch size during each training.**Step 3**Train the CNN by forward and back propagations using Equations (15), (21), and (22).**Output**Return 3D imaging result $\tilde{\beta }$ through the trained CNN.


## 4. Experimental Results

In this section, firstly, the range cells containing scattering information are marked by analyzing the MF back signal. Actually, one range cell corresponds to one imaging plane at a fixed range. Secondly, to compare and analyze the resolving ability of the conventional and the MF-TCAI, we evaluate the radiation-filed randomness of the marked imaging planes by both space independence and effective rank. Thirdly, the imaging results of the conventional and MF-TCAI are presented to show the superiority of MF-TCAI in low SNR. Moreover, compared with the MF-TCAI using a linear frequency modulation (LFM) signal, the FH signal based imaging presents higher space independence and smaller reconstruction error. To obtain higher resolution based on MF, a designed CNN is trained and its training effect is validated by the validation data. Under low SNR, the imaging results of MF-TCAI are put into the trained CNN, leading to higher image quality.

The primary parameters used in the simulations are listed in Table 1. The 3D imaging area includes four imaging scenes, which denote the imaging planes in four different ranges. The transmitting signal used in this section is FH.

High-resolution TCAI requires random phase modulation while it may damage the beam formation, and thus reduce the maximum imaging range. The coded aperture modulates the transmitting signal randomly from [0, π], which will strike a balance between high-resolution imaging and working distance [20].

In order to evaluate the accuracy of the imaging results below, mean square error (MSE) is defined as:

(23) $$MSE={\left\|\beta -\hat{\beta }\right\|}_{2}^{2}/{\left\|\beta \right\|}_{2}^{2}$$

### 4.1. Back Signal Analysis

**Figure 5:**
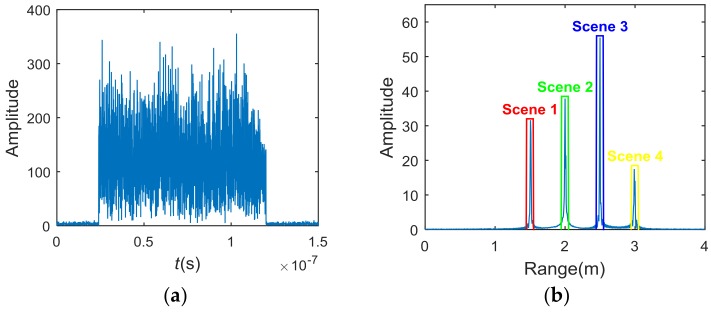
(**a**) The original back signal; (**b**) The back signal after MF.

According to Equation (4), the conventional TCAI imaging reconstructs the target by the original back signal, which is shown in Figure 5a. Then, the original signal is further processed with MF introduced in Section 2.2. As the 3D imaging area contains four scenes or imaging planes, the processed signal in Figure 5b presents four spike pulses around 1.5 m, 2 m, 2.5 m, and 3 m, respectively. As shown in Figure 5b, the spike pulses corresponding to scene 1, 2, 3, and 4 are marked with red, green, blue, and yellow rectangular boxes, respectively. Fortunately, the useful information gathers in the main lobe of spike pulses while the noise is effectively restrained. With different range information located in different spike pulses, it is easy to divide and extract the back signal of different scenes. Using the processed signal, target reconstructions of the four scenes can perform simultaneously under low SNR.

### 4.2. Radiation Field Analysis

**Figure 6:**
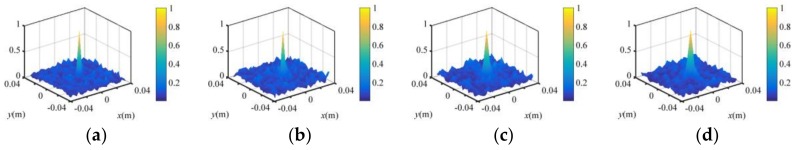

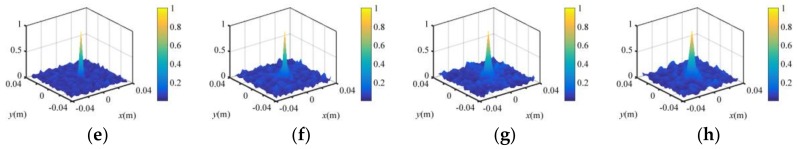
(**a**–**d**) Space independence functions for conventional TCAI corresponding to scenes 1, 2, 3, and 4, respectively; (**e**–**h**) Space independence functions for MF-TCAI corresponding to scenes 1, 2, 3, and 4, respectively.

In this section, we use the space independence function and effective rank [25] to describe the TCAI resolving ability qualitatively and quantitatively, respectively. Figure 6a–d,e–h depict the space independence functions for conventional and MF-TCAI corresponding to different scenes, respectively. The space independence function is inversely proportional to the resolving ability, meaning the thinner the main lobe is, the higher the resolving ability gets. As shown in Figure 6, for both conventional and MF-TCAI, the main lobes of the space independence function grow thicker from scene 1 to scene 4. For the same scene, the main lobes of the MF-TCAI are slightly narrower than those of the conventional one. To verify the observation and analysis, Table 2 reports the effective ranks of the conventional and MF-TCAI for different scenes. The effective rank is proportional to the resolving ability. Whether conventional or MF-TCAI, the effective ranks become smaller from scene 1 to scene 4. Moreover, the effective rank of the MF-TCAI is slightly bigger than that of the conventional one. As scenes 1, 2, 3, and 4 are located at 1.5 m, 2 m, 2.5 m, and 3 m, respectively, the resolving ability is proportional to the range. Furthermore, the MF-TCAI enhances the resolving ability to a certain extent, rather than reducing the resolving ability of the conventional TCAI. Thus, we suppose that MF-TCAI can perform better than the conventional one, even under high SNR.

### 4.3. Initial Imaging Results Based on MF

**Figure 7:**
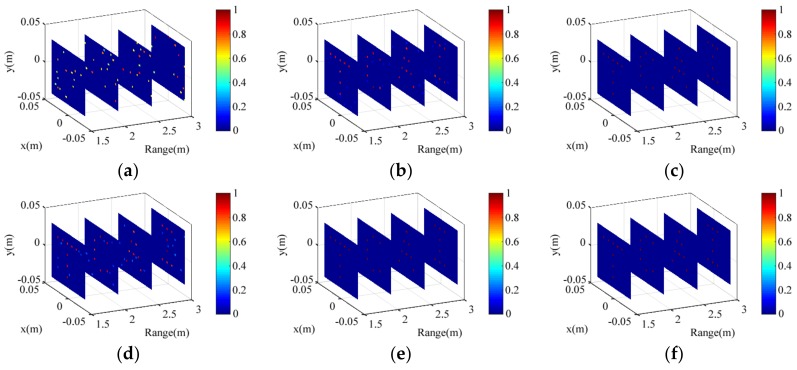
(**a**–**c**) Imaging results of conventional TCAI under different signal-to-noise ratios (SNRs), including: −30 dB, 0 dB, and 30 dB, respectively; (**d**–**f**) Imaging results of MF-TCAI under different SNRs, including: −30 dB, 0 dB, and 30 dB, respectively.

The “T”, “C”, “A”, and “I” shape targets are distributed in the scenes 1, 2, 3, and 4, respectively. The original back signals for radar coded-aperture imaging are simulated by convolution of the scattering coefficients in the scenes and the coded-aperture radiation patterns. The processed signal is obtained by MF. The conventional TCAI and MF-TCAI are based on Equations (4) and (12), respectively. Referring to four scenes in the 3D imaging area, sizes of the reference-signal matrix for conventional TCAI and MF-TCAI are 3600 × 3600 and 900 × 900, respectively. Therefore, the computational complexity of 3D conventional TCAI is much larger than that of MF-TCAI. To compare the imaging ability of the conventional and MF-TCAI, the reconstruction results under different SNRs are shown in Figure 7. In addition, orthogonal matching pursuit (OMP) algorithm is introduced to reconstruct the targets. The experiments are performed on a computer with Intel Core CPU i5-6200U at 2.3 GHz and 8 GB of memory.

Figure 7 illustrates the reconstruction results of conventional TCAI and MF-TCAI under different SNRs. Figure 7a–c describe the results of conventional TCAI under −30 dB, 0 dB, and 30 dB, respectively. In contrast, Figure 7d–f show the results of MF-TCAI under −30 dB, 0 dB, and 30 dB, respectively. When the SNR is −30 dB, the conventional TCAI resolves nothing of the targets, which are shown in Figure 7a. However, as shown in Figure 7d, the MF-TCAI still can resolve the basic information of the target. Under 0 dB, the target reconstruction performs even better with the MF method, as shown in Figure 7e. Unfortunately, as shown in Figure 7b, the conventional TCAI can only reconstruct blurred images with many fake scattering points. When the SNR is 30 dB, both conventional TCAI and MF-TCAI can achieve high-resolution 3D imaging.

**Figure 8:**
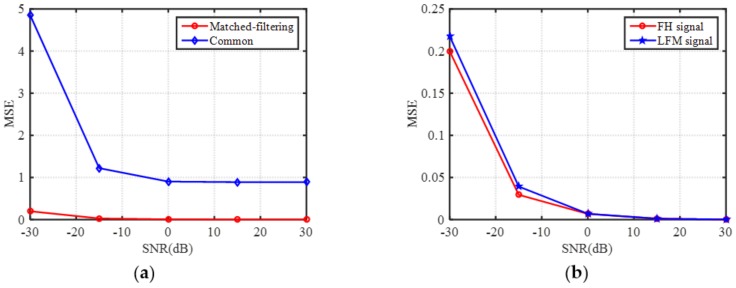
(**a**) Mean square error (MSE) comparisons under different SNRs for, (**a**) common and MF-TCAI, and (**b**) frequency-hopping (FH) and linear frequency modulation (LFM) signal based MF-TCAI.

To further compare the imaging results under 30 dB, Figure 8a gives the MSE comparisons of common TCAI and MF-TCAI under different SNRs. The imaging errors for MF-TCAI are always fewer than those for the common one, especially under lower SNR. Thus, both Figure 7 and Figure 8 show the reconstruction superiority of the MF-TCAI. Moreover, to explain the advantage of using the FH signal, MF-TCAI adopting the LFM signal under the same experiment parameters is operated on the same target. The corresponding effective ranks for scene 1, 2, 3, and 4 are 511, 439, 357, and 283, respectively, which are smaller than those for the FH, as shown in Table 2. It denotes that the spatiotemporal independence of the FH based MF-TCAI is higher than that of the LFM type. Moreover, Figure 8b presents the MSE comparisons under different SNRs for FH and LFM signal based MF-TCAI. Therefore, the FH based MF-TCAI achieve promising higher resolution.

**Figure 9:**
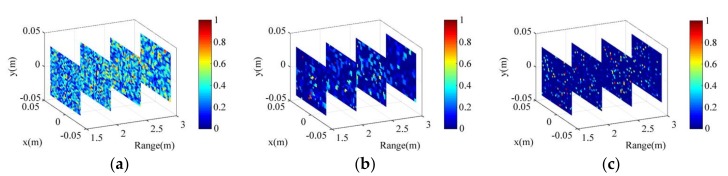

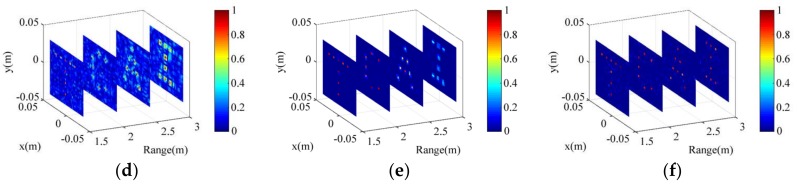
The imaging results of (**a**) sparse Bayesian learning (SBL); (**b**) total variation (TV) regularization; and (**c**) correlation algorithms under −30 dB. The imaging results of (**d**) MF-SBL; (**e**) MF-TV regularization; and (**f**) MF-based correlation algorithms under −30 dB.

Besides OMP, sparse Bayesian learning (SBL) [26] and total variation (TV) [27] algorithm are two other classical algorithms for compressed sensing (CS) for TCAI. With the sparse prior and hierarchical framework, SBL can restrain a certain degree of SNR. TV regularization can recover the target under proper SNR. Besides, successful imaging of TCAI can also be performed with a correlation algorithm [9], which is less sensitive to the presence of clutter. However, present algorithms are all based on Equation (4) without matching pursuit (MF) processing. Equation (4) decides the poor imaging performance under extremely low SNR. Fortunately, all the present algorithms can be improved with MF processing. The imaging model and procedure based on MF are shown in Equation (12) and Algorithm 1, respectively. Under −30 dB, the imaging results of SBL, TV regularization, and correlation algorithms without MF are presented in Figure 9a–c, respectively. Furthermore, the imaging results of SBL, TV regularization, and correlation algorithms with MF are shown in Figure 9d–f, respectively. As shown in Figure 9a–c, under extremely low SNR, all the present algorithms cannot resolve the target. As shown in Figure 9d–f, the basic scattering information of the target can be recognized. Thus, with MF processing, SBL, TV regularization, and correlation algorithms are improved obviously. The advantage of the MF based approach over the present approach is presented clearly.

### 4.4. CNN Training Results

A designed CNN with five layers is trained for further imaging based on the input of the MF-TCAI. Herein, we describe the convolutional kernel of each layer by its size and number, such as $N_{c}\times N_{c}@N_{ci}\times N_{co}$, among which, $N_{c}\times N_{c}$ and $N_{ci}\times N_{co}$ denote the size and number of the kernel, respectively. Moreover, $N_{ci}$ and $N_{co}$ denote the input and output numbers of the corresponding layer, respectively. For the five layers, the kernel parameters are 9×9@1×6, 7×7@6×6, 5×5@6×12, 5×5@12×12, and 3×3@12×1, respectively.

**Figure 10:**
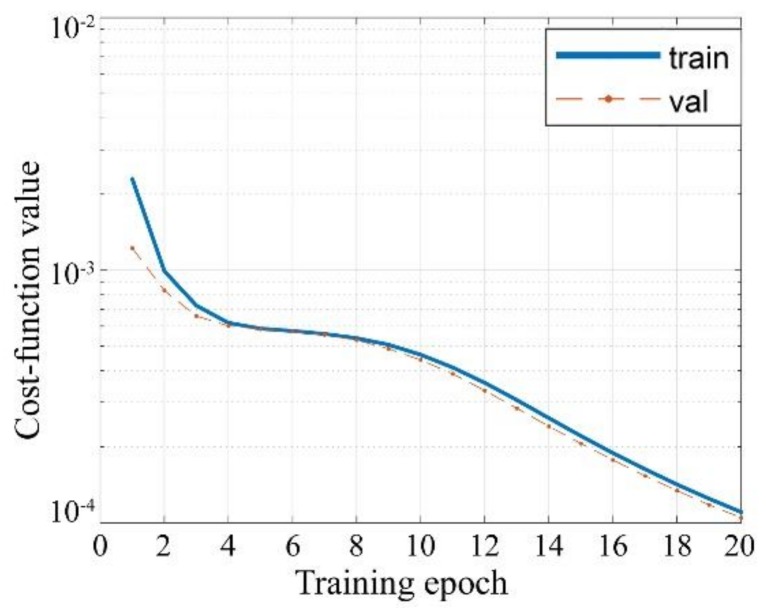
CNN training effect during the learning process.

Weight decay technique is used and the decent coefficient is 0.9. The learning rate is 0.02 for all layers. A total of 75,000 and 25,000 examples are generated, respectively, for training and validation. The generated examples are in different SNRs, including, −30 dB, −15 dB, 0 dB, 15 dB, and 30 dB. The training process lasts for 20 epochs, and the batch size is 20. The cost function not only acts as a feedback for back propagation, but also tells the training effect, which is shown in Figure 10. During the learning process, cost-function values of both the training and validation sets decrease with the increasing epoch, and they finally achieve a quite small imaging error. It indicates that the trained CNN is mature enough for MF-TCAI.

**Figure 11:**
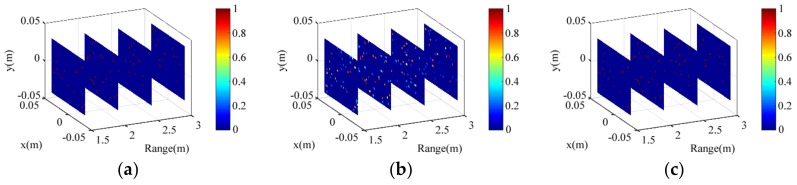
Imaging presentation by the trained CNN for the validation target, including, (**a**) the original target; (**b**) input of the trained CNN: the MF imaging result; and (**c**) output of the trained CNN.

To further verify the training effect, we put a validation set under −30 dB into the trained CNN. Figure 11 presents the CNN input, output, and the original target. Figure 11b presents the CNN input, which is blurred with much fake scattering information. Compared with the original target in Figure 11a, the output image in Figure 11c has reconstructed all the scattering information and removed all the fake points and background noise. Learning from the training sets, the trained CNN not only can filter the disturbing points but also can enhance the true points. Thus, the training effect is verified again.

### 4.5. Further Imaging Results Based on CNN

**Figure 12:**
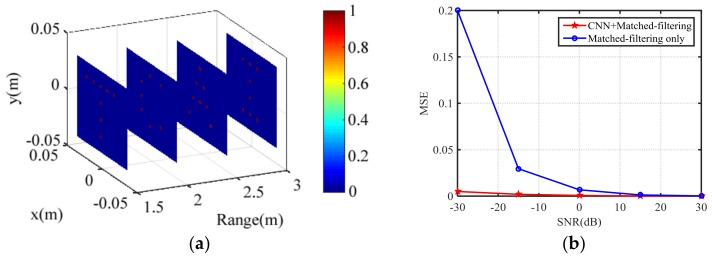
(**a**) CNN-based 3D imaging results under −30 dB; and (**b**) MSE comparisons of MF-TCAI with and without CNN under different SNRs.

As shown in Figure 7d, although the MF-TCAI can reconstruct the basic information of the target under −30 dB, it still contains a lot of background noise, confusing the true target. For further high-resolution imaging, we input the imaging results of Figure 7d into the trained CNN. The CNN output is shown in Figure 12a, which presents that the CNN has removed all the fake information. Moreover, the true scattering points remain and are enhanced. As shown in Figure 12b, to validate the trained CNN quantitatively, we compare the MSE of MF-TCAI with and without CNN under different SNRs. Despite the SNR, the imaging errors processed by CNN are always lower than that processed with simply the MF operation. Especially for −30 dB, the imaging error of CNN based TCAI is much smaller. Therefore, the trained CNN teaches the TCAI how to reconstruct well under low SNR. In conclusion, TCAI based on MF and CNN provides a useful method for 3D high-resolution imaging.

## 5. Conclusions

This paper proposed a 3D imaging method based on MF and CNN to reduce the computational burden and achieve high-resolution imaging for low SNR targets. To divide and extract the useful target information of different ranges, the back signal was processed with MF. Corresponding to the extracted signals for different imaging planes, the frequency-domain reference-signal matrices were constructed. Based on the conventional TCAI, we deduced a MF-TCAI model and introduced the detailed imaging process. Moreover, the designed CNN structure could help the MF-TCAI resolve the low SNR targets more clearly. Finally, numerical experimental results have demonstrated that our imaging method could achieve: (1) high-resolution imaging under low SNR; (2) less computational burden with smaller-sized reference-matrix; (3) stronger resolving ability by using HF rather than LFM; and (4) intelligent imaging with CNN. Moreover, the MF process is not limited to the HF signal, it can also be applied in LFM and stepped-frequency (SF) based TCAI. In conclusion, our proposed 3D TCAI method holds potential applications in areas such as terminal guidance, security checks, etc.

## Figures and Tables

**Table 1:** Primary parameters used in the experiments.

Parameter	Value
Center frequency (*f_c_*)	340 GHz
Bandwidth (B)	20 GHz
Pulse width (*T_p_*)	100 ns
Size of the coded aperture	0.5 m × 0.5 m
Number of the coded-aperture array elements	25 × 25
Sampling frequency (*f*_s_)	25 GHz
Range of Scene 1	1.5 m
Range of Scene 2	2 m
Range of Scene 3	2.5 m
Range of Scene 4	3 m
Size of the grid cell	0.0025 m × 0.0025 m
Number of the convolutional layers	5

**Table 2:** Effective ranks of the conventional and MF-TCAI for different scenes.

	Scene 1	Scene 2	Scene 3	Scene 4
**Conventional TCAI**	568	456	358	279
**MF-TCAI**	581	475	371	290
